# Variant Set Enrichment: an R package to identify disease-associated functional genomic regions

**DOI:** 10.1186/s13040-017-0129-5

**Published:** 2017-02-22

**Authors:** Musaddeque Ahmed, Richard C. Sallari, Haiyang Guo, Jason H. Moore, Housheng Hansen He, Mathieu Lupien

**Affiliations:** 1 Princess Margaret Cancer Centre, University Health Network, Toronto, ON Canada; 2 Department of Medical Biophysics, University of Toronto, Toronto, ON Canada; 3 Massachusetts Institute of Technology (MIT), Cambridge, MA USA; 4 Perelman School of Medicine, University of Pennsylvania, Philadelphia, PA USA; 5 Ontario Institute for Cancer Research, Toronto, ON Canada

**Keywords:** GWAS, Disease, Noncoding region, Enrichment, Regulatory region, AVS

## Abstract

**Background:**

Genetic predispositions to diseases populate the noncoding regions of the human genome. Delineating their functional basis can inform on the mechanisms contributing to disease development. However, this remains a challenge due to the poor characterization of the noncoding genome. Here, we propose an R package that can pinpoint which genomic features are etiologically important based on the genetic predispositions.

**Results:**

Variant Set Enrichment (VSE) is an R package to calculate the enrichment of a set of disease-associated variants across functionally annotated genomic regions, consequently highlighting the mechanisms important in the etiology of the disease studied.

**Conclusions:**

VSE is implemented as an R package and can easily be implemented in any system with R.

**Electronic supplementary material:**

The online version of this article (doi:10.1186/s13040-017-0129-5) contains supplementary material, which is available to authorized users.

## Background

Over 80% of genetic predisposition, namely risk-loci populated by Single Nucleotide Polymorphisms (SNPs), to human diseases identified by Genome Wide Association Studies (GWAS) map to noncoding DNA [1–3]. In other words, most disease-associated SNPs do not directly alter coding sequences. Over the last decade, the functional annotation of the coding and noncoding genome across a wide collection of cell and tissue types benefited from the integration of maps of transcriptional activity from both coding and noncoding transcripts, such as miRNA and long-noncoding RNAs (lncRNAs) as well as chromatin-protein binding profiles, inclusive of transcription factors and epigenetics modifications, and open chromatin. This functional annotation provides a unique opportunity to delineate the functional basis of genetic predispositions to disease.

Here, we present a computational method, named Variant Set Enrichment (VSE) that computes the enrichment/depletion of the set of genetic predisposition for a disease of interest over functional genomic annotations. We previously used a VSE-based approach to identify the enrichment of Breast Cancer (BCa) genetic predispositions at enhancers bound by FOXA1 and ESR1 in breast cancer cells [1]. VSE relies on the set of genetic predispositions and functional annotations; this renders VSE applicable to the study of any genetically inherited disease for which these data are available.

### Implementation

A genetic predisposition (risk-locus) identified by GWAS corresponds to a SNP found on the GWAS array (termed as “tagSNP”) and all SNPs missing from the array but known to be in Linkage Disequilibrium (LD) with the tagSNP (termed as “ldSNP”) [4]. The sum of all genetic predispositions to a particular disease, ie: all the tagSNPs and their ldSNPs constitute the Associated Variant Set (AVS) for that disease.

The identity of risk-loci is user defined, as the cut-off for LD determination is subjected to study preferences. Occasionally, two or more risk-loci for a particular disease can overlap with one another by a common ldSNP. If the common ldSNP overlaps with a functional genomic annotation of interest, the enrichment score calculated by VSE can be inflated because each risk-locus inclusive of this ldSNP would be counted independently. To correct for this possibility, VSE computes a network of all SNPs in which each SNP is represented as a node and the pairwise LD as an edge. Each cluster in the network represents a disjointed locus, as such, a ldSNP is present only in one locus (Additional file 1: Figure S1). VSE then computes the enrichment score of the AVS for each functional genomic annotation of interest in three sequential steps. In the first step, VSE tallies the number of independent risk-loci that overlaps with the functional genomic annotations. Overlapping of a risk-locus is defined as at least one member SNP found within the functional genomic annotation of interest.

This preliminary tallying of AVS may indicate which genomic annotations are functionally related to risk-associated variants, but the overlapping can be affected by size and structure of the AVS. To correct for these biases, VSE, in the second step, computes a null distribution of the overlap tallies that is based on random permutation of AVS. The null AVS is computed by randomly sampling SNPs from a comprehensive pool of tagSNPs present on the GWAS arrays (Illumina Human OmniExpress) and clustering them with their ldSNPs imputed from the 1000 Genome Project Phase III data. When calculating the set of null AVS, VSE makes sure that each set is built in the way that it has identical total number of null loci as the total number of risk-loci in the AVS; and each null locus is matched in size to the corresponding query locus. We defined each null AVS as Matched Random Variant Sets (MRVS).

In the third step, VSE tallies the overlapping of MRVS with the functional genomic annotations of interest. This provides the null distribution to calculate for the enrichment/depletion of the AVS across different functional genomic annotations. To make the enrichment analysis comparable across all functional genomic annotations of interest, MRVS tally is centered at the median and scaled to the standard deviations of the null distribution. The enrichment score is then defined as the number of standard deviations that the overlapping tally deviates from the null overlapping tally median. VSE calculates an exact *P*-value for significance of the enrichment/depletion by fitting a density function to the null distribution derived from the MRVS. The level of significance is corrected for multiple testing using Bonferroni method. The deviation of the null distribution from the normality is tested using Kolmogorov-Smirnov test; and if the distribution deviates, the Box-Cox power transformation is applied on to the null to approach normality.

## Results

**Fig. 1:**
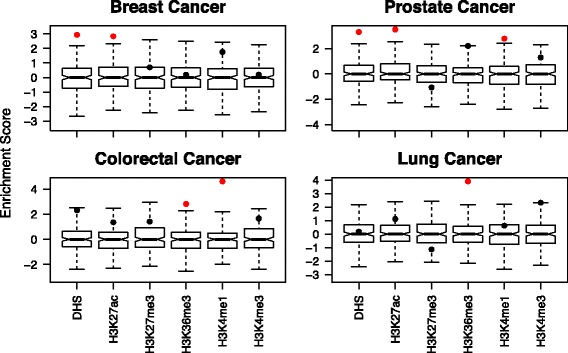
Enrichment of Breast, Prostate, Lung and Colorectal cancer AVS across different genomic maps in cancer-type specific cells. The box and whisker plots show the enrichment score distribution of match null set. The bar inside the box corresponds to the median enrichment score of the null set. The significantly enriched genome regions (Bonferroni corrected *P*-value < 0.01) are marked in red. The histone modifications are profiled in MCF7, LNCaP, A549 and HCT-116/Caco-2 for breast, prostate, lung and colorectal cancer, respectively

The usefulness and impact of VSE is demonstrated by calculating the enrichment of SNPs associated with four cancer types for DNase I Hypersensitivity Sites (DHS) and a set of histone marks profiled genome wide in cancer type relevant cell lines. We compiled 72, 92, 16 and 36 significantly associated SNPs, or tagSNPs, for Prostate Cancer (PRAD), Breast Cancer (BCa), Lung Cancer (LUAD) and Colorectal Cancer (COAD) respectively from NHGRI catalog [5]. We computed the LD structure by finding all SNPs in the European population from the 1000 Genome Project that are in LD with the tagSNPs with r2 ≥ 0.8 [6]. The DNase-seq and ChIP-seq data for H3K4me1 (enhancer), H3K4me3 (promoter), H3K27ac (enhancer and promoter), H3K36me3 (gene body) and H3K27me3 (repressive region) for each of MCF7 (BCa), LNCaP (PRAD), A549 (LUAD) and HCT-116/Caco-2 (COAD) cell lines are compiled from ENCODE data [7] and complemented by data from independent studies [8, 9] (Additional file 1: Table S1). VSE ensures that the distribution of the background is normal by Kolmogorov-Smirnov test and applies transformation if necessary (Additional file 1: Figure S2). Upon performing VSE, the results show that BCa and PRAD AVS are significantly enriched in DHS and regions with H3K27ac mark found in breast and prostate cancer cells, respectively (Fig. 1; Additional file 1: Figures S3 and S4). On the other hand, SNPs associated with LUAD are enriched in regions with H3K36me3 mark only (Fig. 1). In our cross-validation analysis, we observed that the enrichment of an AVS is cancer-type specific, e.g., BCa AVS is enriched in DHS only in MCF7 cells, not in other cells (Additional file 1: Figure S5). The enrichment of distinct cancer AVS across different functional genomic regions argues for a unique biology affected by genetic predispositions across cancer types.

## Conclusions

A set of genetic variants that are strongly associated with a particular disease holds clues about the underlying the mechanism of the development of the disease. VSE provides an easy approach to delineate such information by pinpointing the genomic features that are most affected by the genetic predispositions of that particular disease. In our preliminary analysis, we demonstrate that the genetic variants associated with prostate cancer and breast cancer are significantly over-represented in regulatory regions, while the variants associated with the lung cancer are enriched in coding regions. VSE can be easily implemented in R in any platform. A usage vignette is available in the VSE webspage in CRAN repository and also found in the Additional file 2.

## Additional files

Additional file 1: VSE Practical: Pan-cancer enrichment analysis of genetic predispositions over histone marks. (DOCX 1999 kb)
Additional file 2: VSE Vignette. (PDF 305 kb)

## Abbreviations

AVS: Associated variant set
BCa: Breast cancer
COAD: Colorectal adenocarninoma
DHS: DNaseI hypersensitivity site
GWAS: Genomewide Association Study
LD: Linkage Disequilibrium
lncRNAs: Long-noncoding RNAs
LUAD: Lung adenocarcinoma
MRVS: Matched random variant sets
PRAD: Prostate adenomcarcinoma
SNP: Single nucleotide polymorphisms
VSE: Variant set enrichment
