# Supplementary material for: Variant Set Enrichment: an R package to identify disease-associated functional genomic regions
Source: BioData Min. 2017 Feb 22;10:9. doi: 10.1186/s13040-017-0129-5 (PMC5320724; doi:10.1186/s13040-017-0129-5)
Supplement: Additional file 1: — VSE Practical: Pan-cancer enrichment analysis of genetic predispositions over histone marks. (DOCX 1999 kb) [file 13040_2017_129_MOESM1_ESM.docx]

**VSE Practical: Pan-cancer enrichment analysis of genetic predispositions over histone marks**

**1. Background**

This supplementary document demonstrates a practical example of VSE performance to analyze the enrichment of four cancer type Associated Variant Set (AVS) across genomic maps of DNase I Hypersensitivity Sites (DHS) and several histone modifications. The analysis is performed to predict if any functional genomic region defined by DHS or histone modifications is commonly perturbed or not across different cancer types by genetic predispositions.

**2. Method**

VSE requires a list of all SNPs including the tagSNPs and SNPs that are in LD with the tagSNPs associated with a particular disease and a list of genomic regions of interest. VSE has been developed as an R package (<https://cran.r-project.org/web/packages/VSE/index.html>). A detailed method on how to run is available in the package vignette. In our case study, we focused on breast, prostate, lung and colorectal cancer since genetic predispositions for these cancers have been identified in Genome-Wide Association Studies (GWAS) and Chromatin Immunoprecipitation-sequencing (ChIP-seq) for histone modifications are available in relevant cell lines.

**2.1. Data Collection**

The tagSNPs are curated from NHGRI database with a p-value cutoff of 5e-8. We obtained 92, 72, 36 and 16 tagSNPs for breast, prostate, lung and colorectal cancer, respectively. For the genomic regions, we curated the bed files for H3K4me1, H3K4me3, H3K27me3, H3K27ac and H3K36me3 from various sources (Table 1). These were complemented with DNase-seq data providing the coordinate of DHS.

**2.2. ChIP-seq Quality Control**

The ChIP-seq quality is important to avoid false results. Each ChIP-seq data was controlled for quality using the same criteria. The R Bioconductor package ChIPQC (Carroll *et al.*, 2014) was used to check the quality of each ChIP-seq data. The signal tracks were loaded in Integrative Genomic Browser to visually check the quality of the data. We recommend every user to confirm the good quality of ChIP-seq data in order to obtain high confidence in VSE results.

Table S1: Sources used for genomic region compilation. DHS, DNase Hypersensitive Sites. GSE accession number refers to the dataset available from NCBI Gene Expression Omnibus.

|  |  | **DHS** | **H3K4me1** | **H3K4me3** | **H3K27me3** | **H3K36me3** |
| --- | --- | --- | --- | --- | --- | --- |
| **Prostate Cancer** | Cell line | LNCaP | LNCaP | LNCaP | LNCaP | LNCaP |
|  | Source | ENCODE | GSE47220 | ENCODE | GSE39459 | GSE35829 |
| **Breast Cancer** | Cell line | MCF7 | MCF7 | MCF7 | MCF7 | MCF7 |
|  | Source | ENCODE | (Taberlay *et al.*, 2014) | ENCODE | ENCODE | ENCODE |
| **Colorectal Cancer** | Cell line | HCT-116 | HCT-116 | HCT-116 | Caco-2 | Caco-2 |
|  | Source | ENCODE | ENCODE | ENCODE | ENCODE | ENCODE |
| **Lung Cancer** | Cell line | A549 | A549 | A549 | A549 | A549 |
|  | Source | ENCODE | ENCODE | ENCODE | ENCODE | ENCODE |

The H3K27ac peaks were curated from Hazelett et al. (Hazelett *et al.*, 2014) for prostate cancer and from the ENCODE for the other cancer types.

**2.3. LD SNP calculation**

The easiest and recommended way to calculate the LD blocks is to use the webtool http://raggr.usc.edu. We uploaded our list of SNPs and computed the LD blocks using 1000 Genome Project, Phase III, Oct 2014, Hg19 data, All European population with default setting for other parameters were used.

**2.4. Running VSE**

VSE was run using the R package “VSE”, available in the CRAN repository. To install VSE, type the following command in R:

install.packages(“VSE”)

The usage instruction is elaborated in the vignette.

VSE computes the Associated Variant Sets (AVS) from the list of tag SNPs and their associated LD SNPs provided. It first constructs a network of all SNPs in which each SNP represents a node and their LD associate represents the edge. Each disjoint neighborhood in the network represents a disjoint LD block. VSE then computes Matched Random Variant Sets (MRVS) from 1000 Genome Project genotyping data using the function makeMRVS (Figure 1). The enrichment calculation is critical of the normality of distribution of MRVS intersecting with provided genomic regions. Hence, VSE checks for normality of the MRVS intersecting with each genomic region using Kolmogorv-Smirnov test. A p-value of 1 from the Kolmogorov-Smirnov test indicates a perfectly normal distribution. To achieve a distribution as normal as possible, VSE finds the lambda value for a Box-cox power transformation that gives the P-value for the normality test as close to 1 as possible. VSE then power transforms the null distribution using that lambda (Figure 2). Once the significance of enrichment of AVS across all genomic features was calculated, the p-values will be adjusted for multiple testing using Bonferroni correction method.

**2.5. Caution**

There are certain factors that the user needs to be careful about:

1. VSE is sensitive to the number of tagSNPs. From our trial and error tests, too low number of tagSNPs (below 15) provides imprecise result.
2. Make sure that you use the same r2 cutoff that you used to determine your ldSNPs.
3. The size of the null sets is critical to the calculation of the enrichment. This is because fewer MRVSs cannot accurately model the null distribution. Higher the MRVSs, more statistically accurate the calculations. We recommend a null size between 500 and 1000. The computing time depends heavily on the number of null size and may not be compensated by reducing AVS size or number of genomic features. In our analysis, we chose a size of 500 MRVSs. The size can be set using bgSize parameter in makeMRVS function.

**3. Results**

VSE first creates an Associated Variant Set (AVS) from the list of tag SNPs and their LD SNPs. It then tallies the MRVS and AVS overlaps and calculates the significance of the enrichment.

For this analysis, we observed that the Breast Cancer AVS is enriched in open chromatin (DHS) and H3K27ac in MCF7 cells; prostate cancer AVS is highly enriched in open chromatin, H3K27ac and H3K4me1 marked regions in LNCaP cells; lung cancer AVS in enriched in H3K36me3 marked regions in A549 cells; and colorectal cancer AVS is enriched in H3K36me3 and H3K4me1 marked regions in HCT116 cells (Figure 3). The intersection heatmap outputted by VSE (output.matrix.pdf) is useful to pinpoint which LD block intersects with what genomic feature (Figure 4).

**4. Cross validation**

It is important that a disease specific AVS is matched with a genomic region specific to that disease. The enrichment of an AVS in a particular genomic feature could be cell specific. To validate the cell specificity, we tested the enrichment of each cancer type AVS in the respective most enriched regions across all cell types. For example, since breast cancer AVS is most enriched in DHS in MCF7 cells (Figure 3), we tested breast cancer AVS in DHS regions from A549 (lung cancer), HCT116 (colorectal cancer), MCF7 (breast cancer) and LNCaP (prostate cancer) cells. We observed that the breast cancer AVS is significant enrichment in DHS only from MCF7, not from other cells. Similarly, we tested prostate cancer AVS across DHS regions from all cells, lung cancer AVS across H3K36me3 marked regions from all cells and colorectal cancer AVS across H3K4me1 marked regions from all cells. In each analysis, the enrichment is observed only in matched cells (Figure 5).

Figure S1: A simplified schematic diagram of different steps performed by VSE. LD, Linkage Disequilibrium.


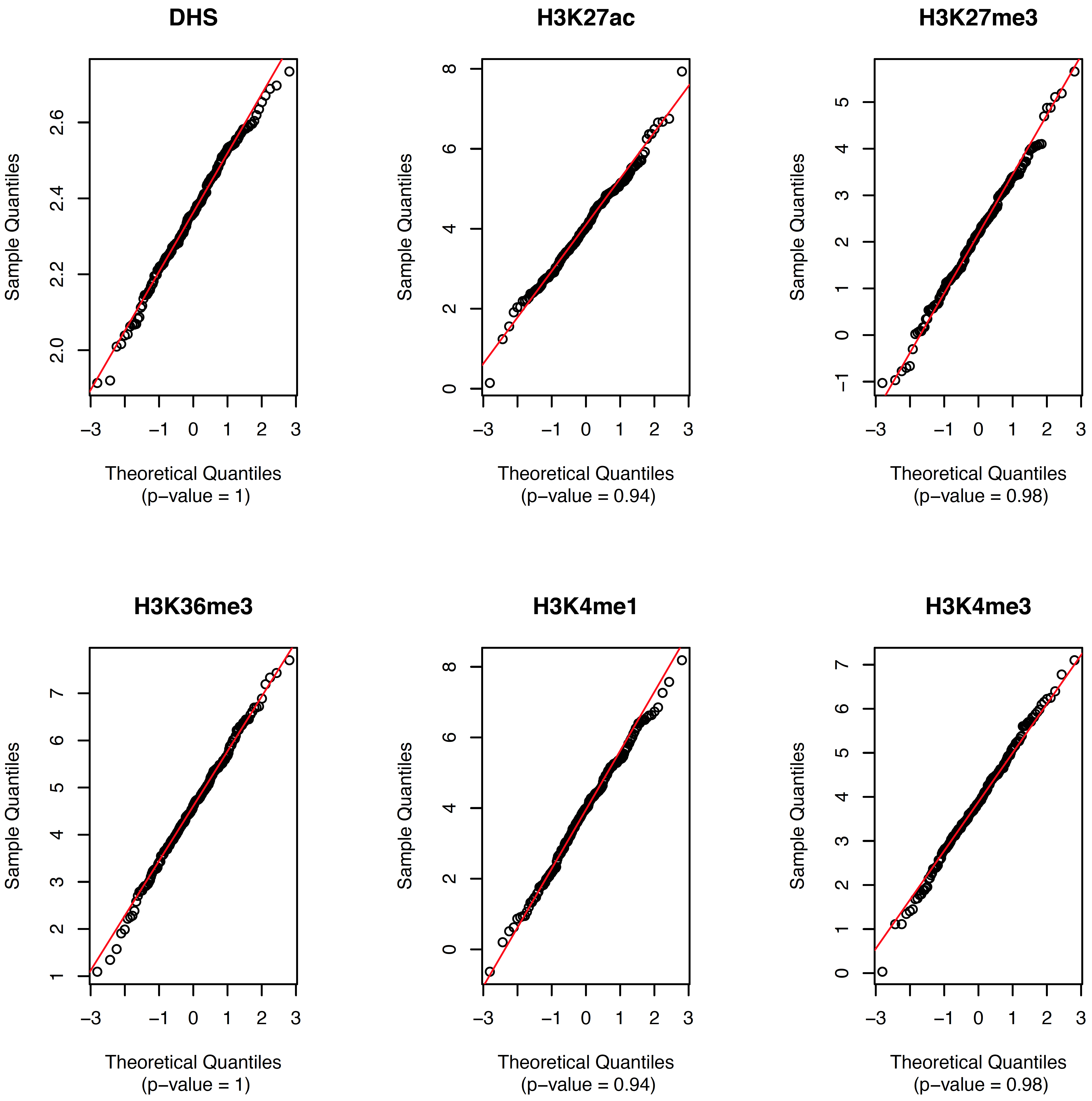


Figure S2: QQ plot of distribution of breast cancer MRVS intersection with six sets of genomic regions provided. DHS and histone marks are from MCF7 cells. The p-values are calculated using Kolmogorov-Smirnov test. A p-value of 1 indicates a normal distribution.


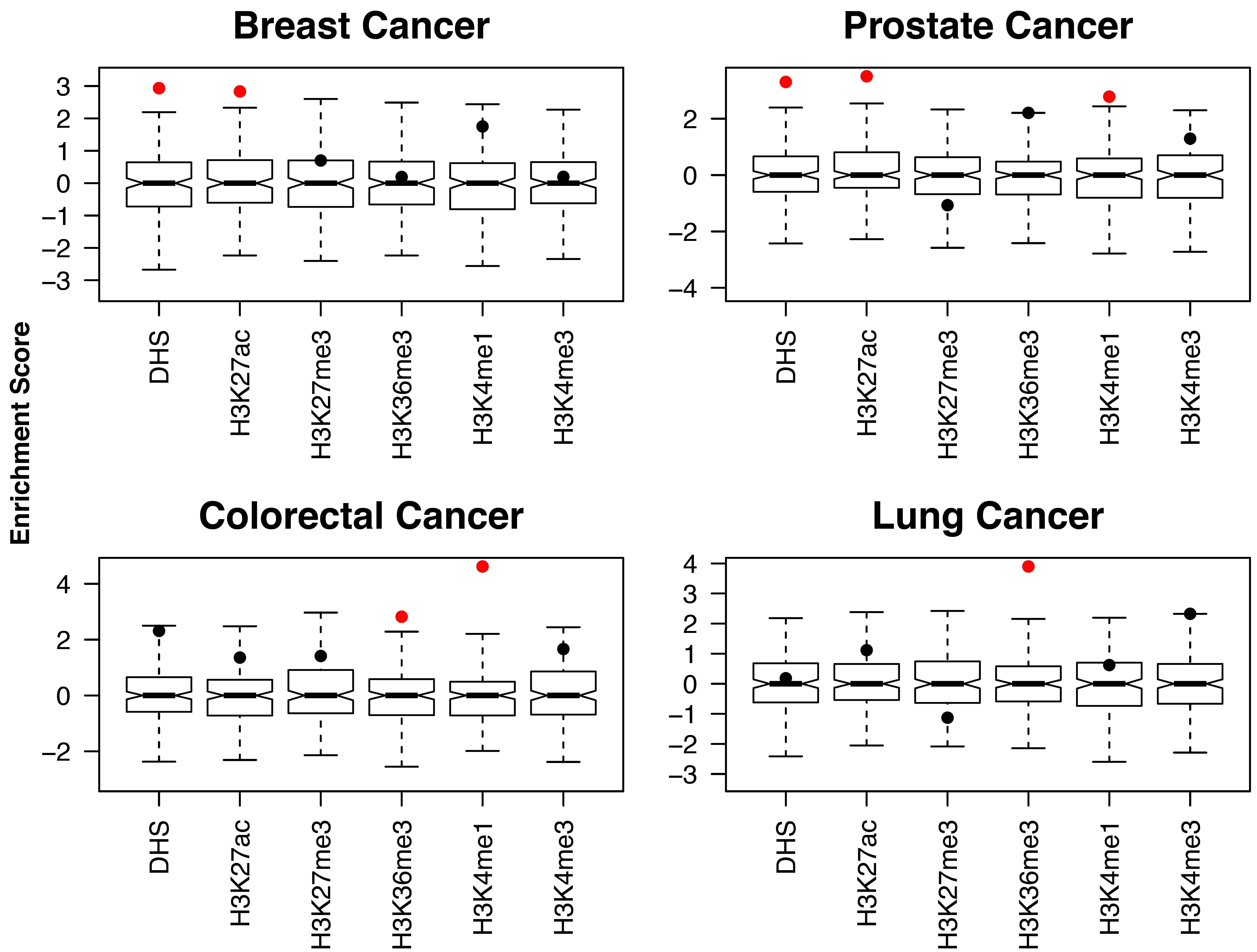


Figure S3: Enrichment of breast cancer, prostate cancer, lung cancer and colorectal cancer associated variant sets across DHS and genomic regions with different histone marks. The box and whisker plots show the enrichment score distribution of match null set. The bar inside the box corresponds to the median enrichment score of the null set. The significantly enriched genome regions (Bonferroni corrected P-value < 0.05) are marked in red.


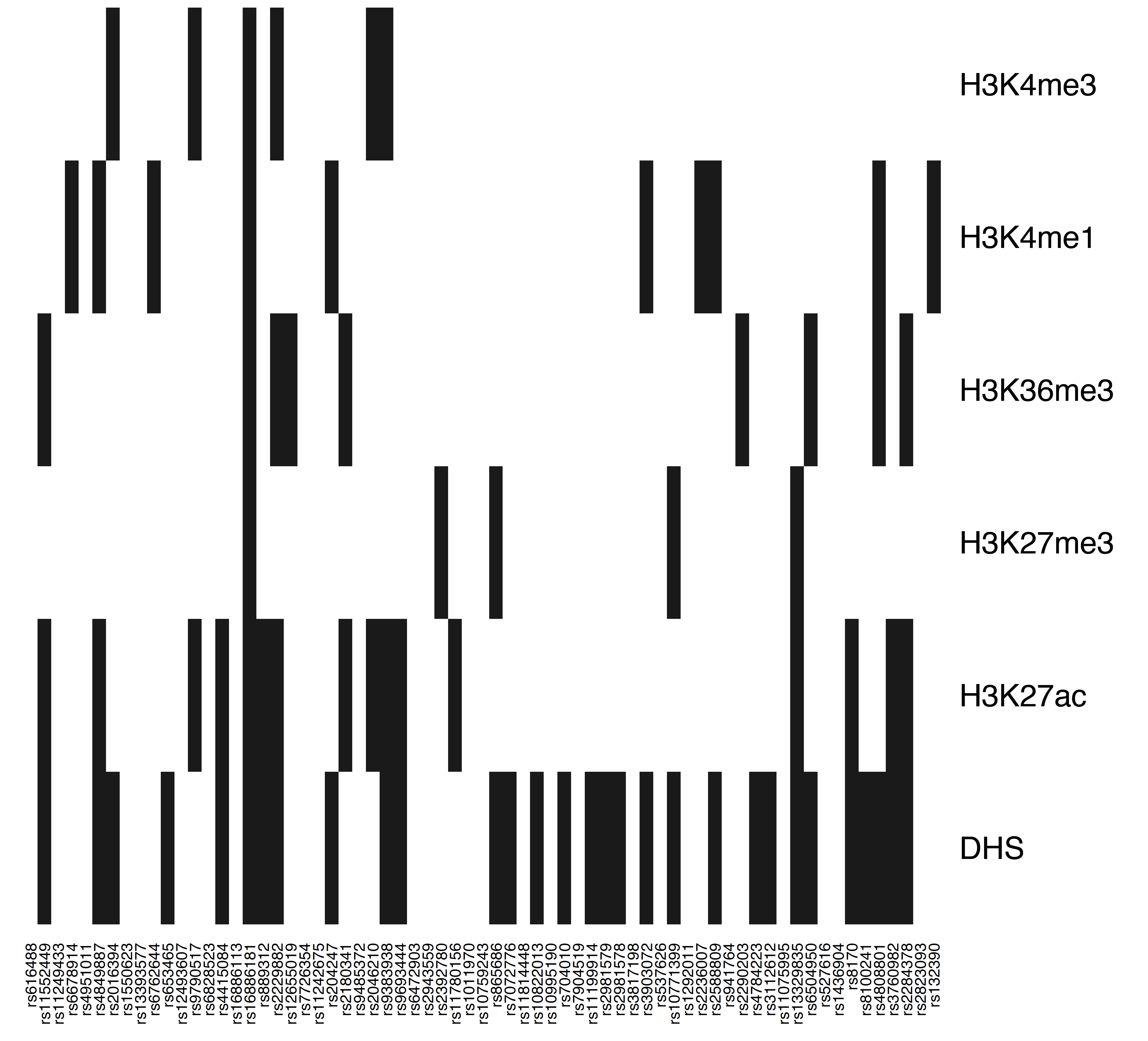


Figure S4: Visualization of overlapping of breast cancer AVS with DHS and histone marks profiled in MCF7.


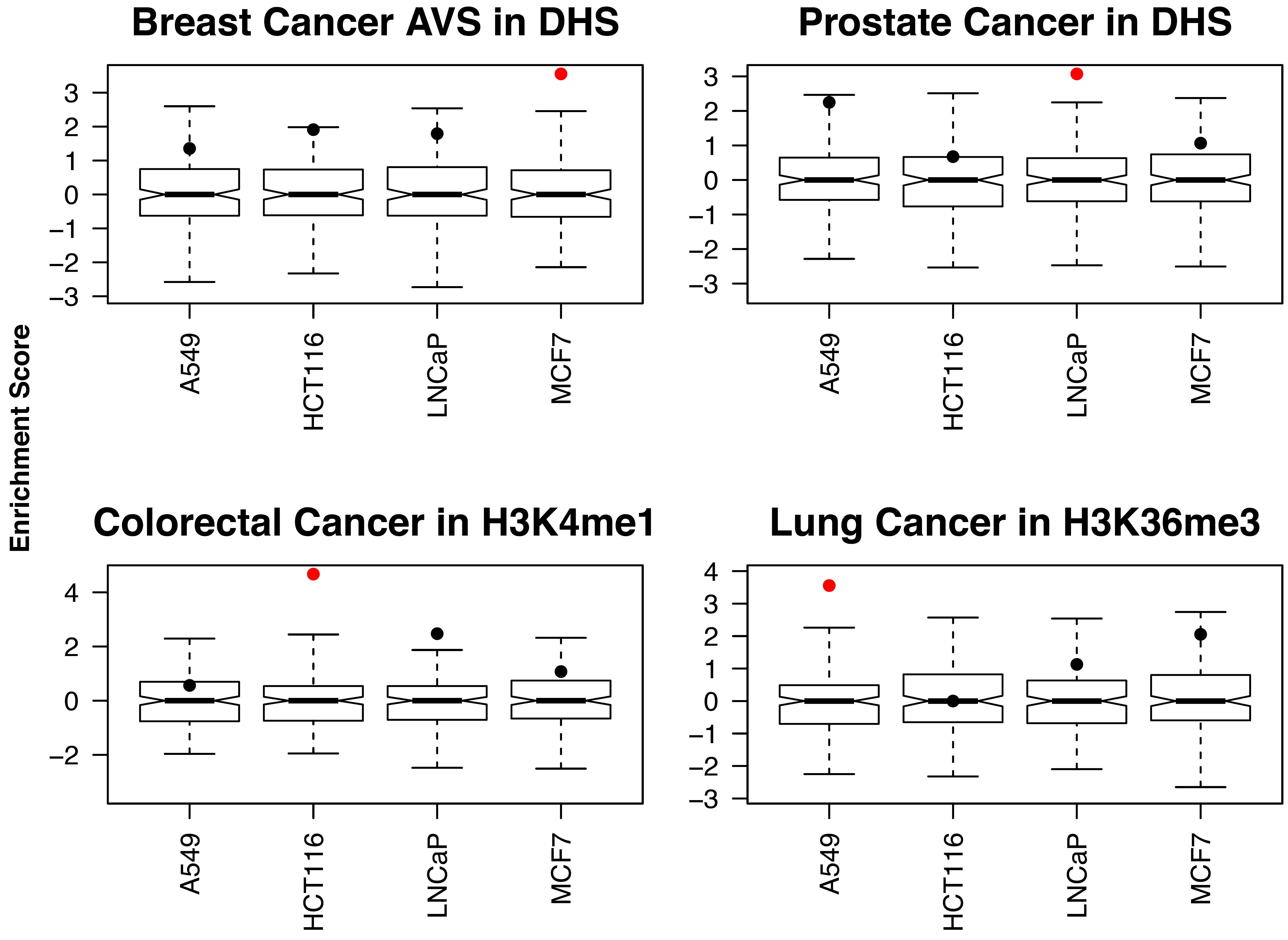


Figure S5: Cross validation of cell specific enrichment of cancer associated variant sets. The box and whisker plots show the enrichment score distribution of match null set. The bar inside the box corresponds to the median enrichment score of the null set. The significantly enriched genome regions (Bonferroni corrected P-value < 0.01) are marked in red.

***Reference***

Carroll,T.S. *et al.* (2014) Impact of artifact removal on ChIP quality metrics in ChIP-seq and ChIP-exo data. *Bioinforma. Comput. Biol.*, **5**, 75.

Hazelett,D.J. *et al.* (2014) Comprehensive Functional Annotation of 77 Prostate Cancer Risk Loci. *PLoS Genet.*, **10**, e1004102.

Purcell,S. *et al.* (2007) PLINK: A Tool Set for Whole-Genome Association and Population-Based Linkage Analyses. *Am. J. Hum. Genet.*, **81**, 559–575.

Taberlay,P.C. *et al.* (2014) Reconfiguration of nucleosome-depleted regions at distal regulatory elements accompanies DNA methylation of enhancers and insulators in cancer. *Genome Res.*, **24**, 1421–1432.

The 1000 Genomes Project Consortium (2012) An integrated map of genetic variation from 1,092 human genomes. *Nature*, **491**, 56–65.
