# Supplementary material for: Variant Set Enrichment: an R package to identify disease-associated functional genomic regions
Source: BioData Min. 2017 Feb 22;10:9. doi: 10.1186/s13040-017-0129-5 (PMC5320724; doi:10.1186/s13040-017-0129-5)
Supplement: Additional file 2: — VSE Vignette. (PDF 305 kb) [file 13040_2017_129_MOESM2_ESM.pdf]

Variant Set Enrichment - Example run for Breast Cancer AVS against DHS and histone marks in MCF7

Musaddeque Ahmed musa.ahmed@utoronto.ca

2016-03-23

VSE calculates the enrichment of associated variant set (AVS) for an array of genomic regions. The AVS is the collection of disjoint LD blocks computed from a list of disease associated SNPs and their linked (LD) SNPs. VSE generates a null distribution of matched random variant sets (MRVSs) from 1000 Genome Project Phase III data that are identical to AVS, LD block by block. It then computes the enrichment of AVS intersecting with user provided genomic features (e.g., histone marks or transcription factor binding sites) compared with the null distribution.

Introduction

The Genomewide association studies have blossomed recently. These studies have outputted genetic variants that are significantly associated to the patients with the related condition. These genetic variants are genetic predispositions to that particular condition. It is extremely important to check if the genetic predispositions for a particular disease are enriched in any particular genomic features. VSE computes exactly that. With a given list of genetic predisitions along with the closely linked SNPs and a list of genomic features, VSE computes if the AVS is significantly enriched in any given genomic feature. This provides a great primer on what features are etiological important for that disease.

Installation

We recommend the use of the latest R version (downloadable from http://cran.r-project.org/mirrors.html). You must also install two Bioconductor packages - `GenomicRanges` and `IRanges`.

```
#Install bioconductor packages
source("http://bioconductor.org/biocLite.R")
biocLite("GenomicRanges")
biocLite("IRanges")

#Install VSE
install.packages("VSE")
```

Please make sure all related packages are up-to-date. If any package is not, you will get an error message during installation of VSE. Installing the missing/outdated package should solve the problem.

Running VSE

As an example, we are going to analyze the enrichment of Breast Cancer AVS across histone marks available for BCa cells, MCF7. We have collected ChIP-seq data for H3K4me1, H3K27me3, H3K27ac, H3K36me3, H3K4me3 and DNAase-seq (DHS) for MCF7 from ENCODE project. We have also extracted the genetic predispositions (tag SNPs) for BCa from GWAS catalog. The following vignette will show step by step instruction to analyze the enrichment of BCa AVS in the above-mentioned histone marks in MCF7.

Cheatsheet

The following block of code will output the VSE analysis for Breast Cancer example dataset. For details, customization and/or different dataset, please continue reading to subsequent sections.

```
library("VSE")
#Load ld
bca.ld <- loadLd(file.path(system.file("extdata",
                                       "ld_BCa_raggr.csv",
                                       package="VSE")),
                 type="raggr")
#Make AVS
bca.avs <- makeAVS(bca.ld)
#Load MRVSs
load(file.path(system.file("extdata",
                           "bca.mrvs.200.Rda",
                           package="VSE")))
#Downloading sample regions
sampleSheet_path <- loadSampleRegions()
#Loading sample sheet
samples <- read.csv(sampleSheet_path, header=TRUE)
#Run VSE
bca.vse <- variantSetEnrichment(bca.avs, bca.mrvs.200, samples)
#Get summary result
VSESummary(bca.vse)
#Visualize
VSEplot(bca.vse,
        las=2,
        pch=20,
        cex=1,
        cex.main=0.6,
        padj = 0.05,
        main="BCa AVS in MCF7 genomic features")
```

Loading BCa genetic predispositions

The associated SNPs (tag SNPs) for a particular disease can be downloaded from GWAS catalog or other GWAS studies. The tag SNPs are typically representative of their respective LD blocks, and any SNP that are in close linkage disequilibrium has the potential to be causal SNP. Thus, it is important to consider all SNPs that are high LD with the tag SNPs. The easiest and recommended way to calculate the LD blocks is to use the webtool http://raggr.usc.edu. Upload your list of SNP and compute the LD blocks using 1000 Genome Project, Phase III, Oct 2014, Hg19 data, All European population with default setting for other parameters. Save the "Union of results" from the result page and unzip the file. We have done this for BCa tag SNPs obtained from GWAS catalog and got the union result. The resulting file has been provided with this package as an example data. Let's load this data into VSE:

```
library("VSE")
bca.ld <- loadLd(file.path(system.file("extdata", "ld_BCa_raggr.csv", package = "VSE")),
    type = "raggr")
# bca.ld is a GRanges object
bca.ld
```

```
## GRanges object with 2541 ranges and 2 metadata columns:
```

```
##          seqnames                 ranges strand   |       idLd      idTag
##             <Rle>              <IRanges>  <Rle>   |   <factor>   <factor>
##       [1]        1 [ 10566216,  10566216]      *   |   rs616488   rs616488
##       [2]        1 [ 10566273,  10566273]      *   |   rs616402   rs616488
##       [3]        1 [114426002, 114426002]      *   | rs11102694 rs11552449
##       [4]        1 [114429462, 114429462]      *   |  rs2358994 rs11552449
##       [5]        1 [114429516, 114429516]      *   |  rs2358995 rs11552449
##       ...      ...                    ...    ... ...        ...        ...
##    [2537]       21   [16520357, 16520357]      *   | rs34562325  rs2823093
##    [2538]       21   [16520833, 16520833]      *   |  rs2823093  rs2823093
##    [2539]       21   [16520936, 16520936]      *   |  rs2896680  rs2823093
##    [2540]       22   [29621478, 29621478]      *   |   rs132390   rs132390
##    [2541]       22   [29621862, 29621862]      *   |   rs132391   rs132390
##   -------
##   seqinfo: 21 sequences from an unspecified genome; no seqlengths
```

Making AVS

At the first step, a disjoint list of associated variant set (AVS) must be constructed, in which, one SNP is present in only one LD block to avoid inflation of enrichment. The disjoint LD blocks are generated by making a network of all SNPs where each node is a SNP and each EDGE is the linkage disequilibrium. A neighborhood in the network is a disjointed LD block.

```
bca.avs <- makeAVS(bca.ld)
# Check the size of each LD block
avs.size <- avsSize(bca.avs)
head(avs.size)
```

```
##        tagID Size
## 1   rs616488    2
## 2 rs11552449    9
## 3 rs11249433    2
## 4  rs6678914   36
## 5  rs4951011   39
## 6  rs4849887   60
```

```
library(ggplot2)
ggplot(avs.size, aes(x = tagID, y = Size)) + geom_bar(stat = "identity") + coord_flip() +
    theme_classic() + theme(axis.text.y = element_text(size = 5))
```

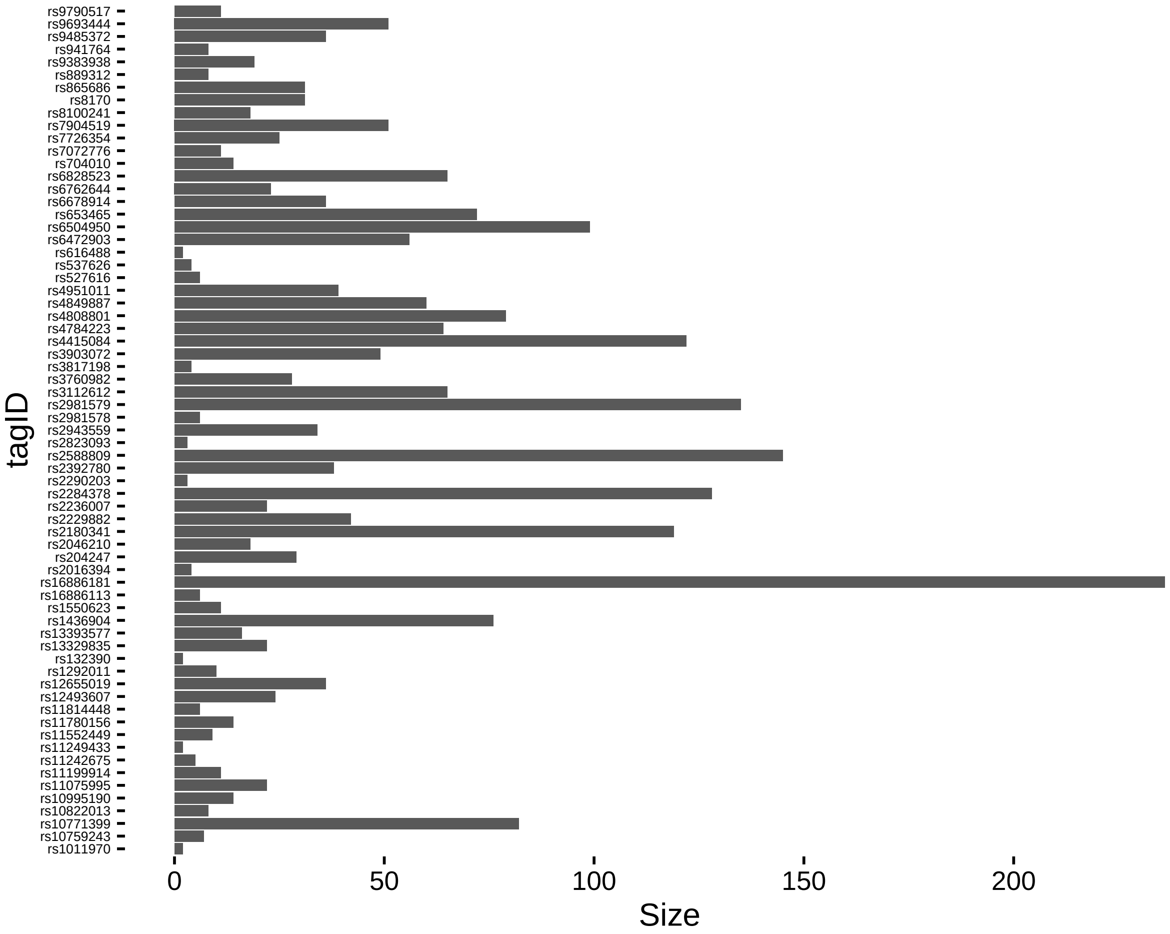

bca.avs is a GRangesList object. It can be converted to dataframe or a bed file for other use.

```
bca.avs
```

```
## GRangesList object of length 67:
## [[1]]
## GRanges object with 2 ranges and 2 metadata columns:
##       seqnames               ranges strand |     idLd    idTag
##          <Rle>            <IRanges>  <Rle> | <factor> <factor>
##   [1]        1 [10566216, 10566216]      * | rs616488 rs616488
##   [2]        1 [10566273, 10566273]      * | rs616402 rs616488
##
## [[2]]
## GRanges object with 9 ranges and 2 metadata columns:
##       seqnames                 ranges strand |       idLd      idTag
##   [1]        1 [114426002, 114426002]      * | rs11102694 rs11552449
##   [2]        1 [114429462, 114429462]      * |  rs2358994 rs11552449
##   [3]        1 [114429516, 114429516]      * |  rs2358995 rs11552449
##   [4]        1 [114431464, 114431464]      * |  rs7547478 rs11552449
##   [5]        1 [114445881, 114445881]      * |  rs7513707 rs11552449
##   [6]        1 [114448390, 114448390]      * | rs11552449 rs11552449
##   [7]        1 [114449663, 114449663]      * |  rs3761936 rs11552449
##   [8]        1 [114449830, 114449830]      * | rs11102701 rs11552449
##   [9]        1 [114450965, 114450965]      * | rs11102702 rs11552449
```

```
##
## [[3]]
## GRanges object with 2 ranges and 2 metadata columns:
##       seqnames                 ranges strand |       idLd      idTag
##    [1]        1 [121213349, 121213349]      * | rs12134101 rs11249433
##    [2]        1 [121280614, 121280614]      * | rs11249433 rs11249433
##
## ...
## <64 more elements>
## -------
## seqinfo: 21 sequences from an unspecified genome; no seqlengths
```

We have also computed the MRVSs for prostate, colorectal and lung cancer. Please read at the bottom of this document to learn how to download them.

Constructing Matched Random Variant Sets(MRVSs)

To compute the enrichment of AVS in genomic features, a null set must be constructed. MRVSs are identical to AVS block by block. The argument bgSize denotes the size of the null. Higher the null size, more accurate the analysis. In typical cases, a background size of 100 is sufficient. This is the most time consuming part, but once the MRVSs are computed for a particular AVS, it can be used for several genomic features. The argument mc.cores denotes the the number of cores to be used for this step.

We have generated, saved and added the MRVS (size = 200) data for BCa AVS with the package as example. You can load it by:

```
# Load MRVS
load(file.path(system.file("extdata", "bca.mrvs.200.Rda", package = "VSE")))
class(bca.mrvs.200)
```

```
## [1] "list"
```

We have also computed the MRVSs for prostate, colorectal and lung cancer. Please read at the bottom of this document to learn how to download them.

If you have an AVS other than the ones provided (e.g., for another disease), you can compute MRVSs yourself. For example, to generate MRVSs for lung cancer AVS:

```
#Download lung cancer AVS
url <- "http://www.hansenhelab.org/VSE/LCa-AVS.RData"
download.file("http://www.hansenhelab.org/VSE/LCa-AVS.RData", destfile="LCa-AVS.RData", method = "curl"
load("LCa-AVS.RData") #Will load sca.avs into environment, which is the AVS for LCa
lca.mrvs.100 <- makeMRVS(sca.avs, bgSize=100, mc.cores = 8)
```

Note for Windows user: You must use mc.cores = 1 as argument for makeMRVS

Since generating mrvs takes considerably longer preiod, it is ideal to save the MRVS for future use. MRVSs are constant for a particular AVS, thus it is not necessary to regenerate for the same AVS.

```
# Save MRVS for future use
save(lca.mrvs.100, file="lca.mrvs.100.Rda")
```

Loading genomic features

Once the AVS and MRVSs are ready, you can do intersection analysis of AVS across a set of genomic features. The genomic features could be peak coordinates from ChIP-seq data or any other genomic coordinates in bed format. For example, we have added the ChIP-seq peak data for 5 histone marks and DNAse-seq data for MCF7 obtained from ENCODE.

The list of genomic features must be prepared in a sample sheet, similar to what used for ChIPQC or DiffBind package. For example, we have added a function `loadSampleRegions()` which will download 6 region coordinates in bed format and a sample sheet from www.hansenhelab.org/VSE/sample_regions/. If you download these files in a location other the default below, please change the path in the `Peaks` column in `sampleSheet.csv`.

```
# Downloading sample regions
sampleSheet_path <- loadSampleRegions()
# Loading sample sheet
samples <- read.csv(sampleSheet_path, header = TRUE)
samples
```

```
##   X SampleID Tissue   Factor Replicate bamReads
## 1 1      DHS Breast      DHS         1       NA
## 2 2  H3K27ac Breast  H3K27ac         1       NA
## 3 3 H3K27me3 Breast H3K27me3         1       NA
## 4 4 H3K36me3 Breast H3K36me3         1       NA
## 5 5  H3K4me1 Breast  H3K4me1         1       NA
## 6 6  H3K4me3 Breast  H3K4me3         1       NA
##                                                                                Peaks
## 1      /var/folders/4z/b97_jpxs3gq6h9m6wcfz98000000gn/T//RtmpghvtiB/VSE_samples/DHS.bed
## 2  /var/folders/4z/b97_jpxs3gq6h9m6wcfz98000000gn/T//RtmpghvtiB/VSE_samples/H3K27ac.bed
## 3 /var/folders/4z/b97_jpxs3gq6h9m6wcfz98000000gn/T//RtmpghvtiB/VSE_samples/H3K27me3.bed
## 4 /var/folders/4z/b97_jpxs3gq6h9m6wcfz98000000gn/T//RtmpghvtiB/VSE_samples/H3K36me3.bed
## 5  /var/folders/4z/b97_jpxs3gq6h9m6wcfz98000000gn/T//RtmpghvtiB/VSE_samples/H3K4me1.bed
## 6  /var/folders/4z/b97_jpxs3gq6h9m6wcfz98000000gn/T//RtmpghvtiB/VSE_samples/H3K4me3.bed
```

Check intersection of each LD block across genomic features

The intersection of each LD block in the AVS with the genomic features can be visualized.

```
bca.intersect <- intersectMatrix(bca.avs, regions = samples, col = c("white",
    "grey10"), scale = "none", margins = c(5, 5), cexRow = 1, cexCol = 0.5,
    Rowv = NA, Colv = NA)
```

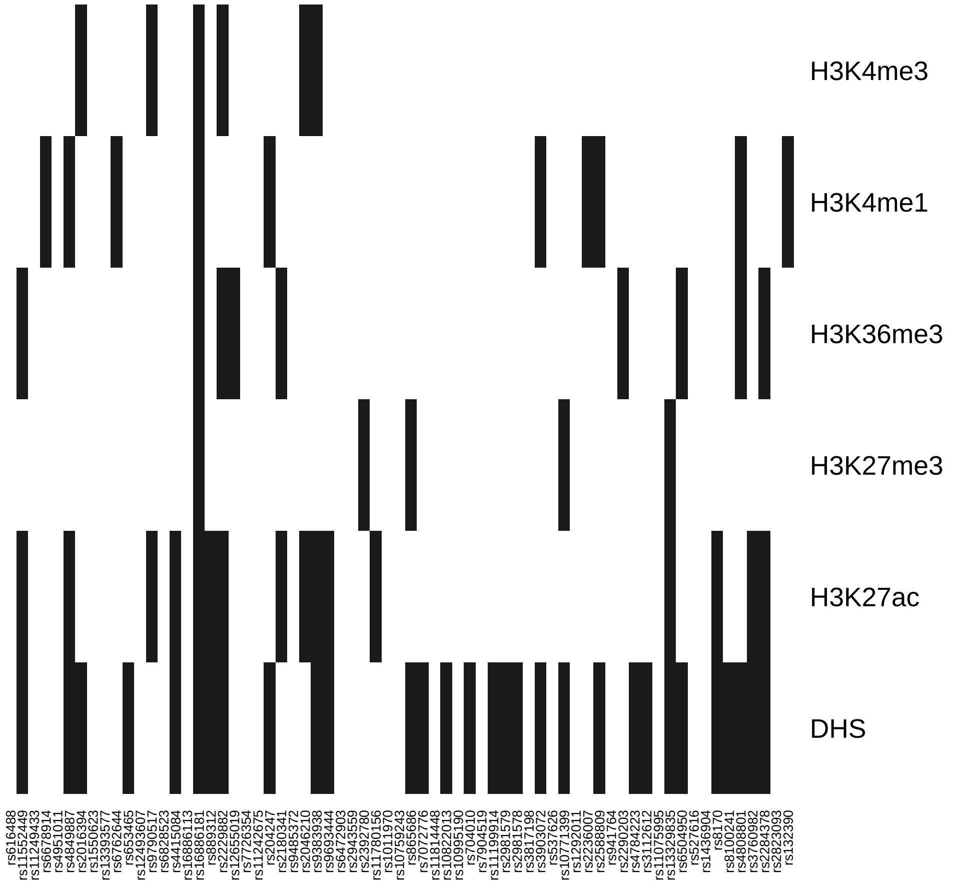


```
bca.intersect
```

```
##           rs616488 rs11552449 rs11249433 rs6678914 rs4951011 rs4849887
## DHS              0          1          0         0         0         1
## H3K27ac          0          1          0         0         0         1
## H3K27me3         0          0          0         0         0         0
## H3K36me3         0          1          0         0         0         0
## H3K4me1          0          0          0         1         0         1
## H3K4me3          0          0          0         0         0         0
##           rs2016394 rs1550623 rs13393577 rs6762644 rs653465 rs12493607
## DHS               1         0          0         0        1          0
## H3K27ac           0         0          0         0        0          0
## H3K27me3          0         0          0         0        0          0
## H3K36me3          0         0          0         0        0          0
## H3K4me1           0         0          0         1        0          0
## H3K4me3           1         0          0         0        0          0
##           rs9790517 rs6828523 rs4415084 rs16886113 rs16886181 rs889312
## DHS               0         0         1          0          1        1
## H3K27ac           1         0         1          0          1        1
## H3K27me3          0         0         0          0          1        0
## H3K36me3          0         0         0          0          1        0
## H3K4me1           0         0         0          0          1        0
## H3K4me3           1         0         0          0          1        0
##           rs2229882 rs12655019 rs7726354 rs11242675 rs204247 rs2180341
## DHS               1          0         0          0        1         0
```

```
## H3K27ac          1          0         0          0        0         1
## H3K27me3         0          0         0          0        0         0
## H3K36me3         1          1         0          0        0         1
## H3K4me1          0          0         0          0        1         0
## H3K4me3          1          0         0          0        0         0
##          rs9485372 rs2046210 rs9383938 rs9693444 rs6472903 rs2943559
## DHS              0         0         1         1         0         0
## H3K27ac          0         1         1         1         0         0
## H3K27me3         0         0         0         0         0         0
## H3K36me3         0         0         0         0         0         0
## H3K4me1          0         0         0         0         0         0
## H3K4me3          0         1         1         0         0         0
##          rs2392780 rs11780156 rs1011970 rs10759243 rs865686 rs7072776
## DHS              0          0         0          0        1         1
## H3K27ac          0          1         0          0        0         0
## H3K27me3         1          0         0          0        1         0
## H3K36me3         0          0         0          0        0         0
## H3K4me1          0          0         0          0        0         0
## H3K4me3          0          0         0          0        0         0
##          rs11814448 rs10822013 rs10995190 rs704010 rs7904519 rs11199914
## DHS               0          1          0        1         0          1
## H3K27ac           0          0          0        0         0          0
## H3K27me3          0          0          0        0         0          0
## H3K36me3          0          0          0        0         0          0
## H3K4me1           0          0          0        0         0          0
## H3K4me3           0          0          0        0         0          0
##          rs2981579 rs2981578 rs3817198 rs3903072 rs537626 rs10771399
## DHS              1         1         0         1        0          1
## H3K27ac          0         0         0         0        0          0
## H3K27me3         0         0         0         0        0          1
## H3K36me3         0         0         0         0        0          0
## H3K4me1          0         0         0         1        0          0
## H3K4me3          0         0         0         0        0          0
##          rs1292011 rs2236007 rs2588809 rs941764 rs2290203 rs4784223
## DHS              0         0         1        0         0         1
## H3K27ac          0         0         0        0         0         0
## H3K27me3         0         0         0        0         0         0
## H3K36me3         0         0         0        0         1         0
## H3K4me1          0         1         1        0         0         0
## H3K4me3          0         0         0        0         0         0
##          rs3112612 rs11075995 rs13329835 rs6504950 rs527616 rs1436904
## DHS              1          0          1         1        0         0
## H3K27ac          0          0          1         0        0         0
## H3K27me3         0          0          1         0        0         0
## H3K36me3         0          0          0         1        0         0
## H3K4me1          0          0          0         0        0         0
## H3K4me3          0          0          0         0        0         0
##          rs8170 rs8100241 rs4808801 rs3760982 rs2284378 rs2823093 rs132390
## DHS           1         1         1         1         1         0        0
## H3K27ac       1         0         0         1         1         0        0
## H3K27me3      0         0         0         0         0         0        0
## H3K36me3      0         0         1         0         1         0        0
## H3K4me1       0         0         1         0         0         0        1
## H3K4me3       0         0         0         0         0         0        0
```

Calculate enrichment

To calculate enrichment, variantSetEnrichment function requires three arguments - an AVS GRangesList object outputted by makeAVS function, a bca.mrvs list outputted by makeMRVS function, and a region dataframe with genomic features. The enrichment can be run by:

```
bca.vse <- variantSetEnrichment(bca.avs, bca.mrvs.200, samples)
```

variantSetEnrichment output is a list of three matrices, in which, the second matrix is the normalized null distribution. It is essential that the null distribution is a normal distribution. Normality of the null can be confirmed using a QQ-plot.

```
par.original <- par(no.readonly = TRUE)
par(mfrow = c(ceiling(length(samples$Peaks)/3), 3), mai = c(1, 1, 0.5, 0.1))
VSEqq(bca.vse)
```

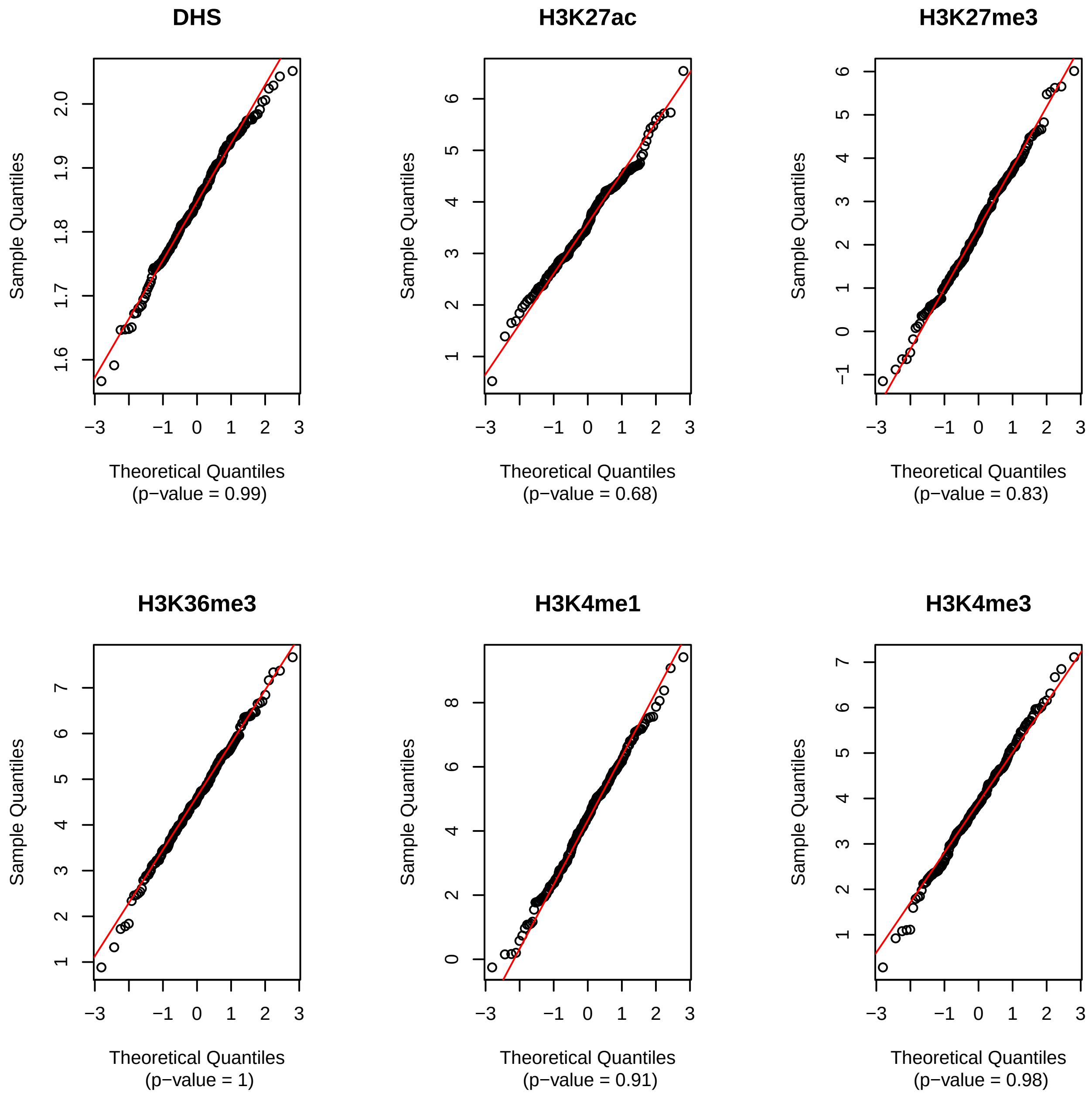


```
par(par.original)
```

The normality of the distribution is confirmed using Kolmogorv-Smirnov test with the null hypothesis of that the null distribution is different than a normal distribution. The significance of the test is outputted underneath the plots. Any distribution with significant difference (p-value < 0.05) should not be considered for the subsequent analysis. However, VSE transforms the null distribution by Box-cox power transformation which approaches the normality of the data. It is very unlikely that the null distribution will not be normal if the background size is sufficiently large (over 100).

Save results

The VSE result can be summarized by:

```r
bca.vse.res <- VSESummary(bca.vse)
bca.vse.res
```

```
##             region avs  enrichment      p.value  p.adjusted
## DHS            DHS  30  3.16545062 0.0008410123 0.005046074
## H3K27ac    H3K27ac  16  2.65753321 0.0044547855 0.026728713
## H3K27me3 H3K27me3   5  1.08442481 0.1456020692 0.873612415
## H3K36me3 H3K36me3   9  0.09606206 0.4625163235 1.000000000
## H3K4me1    H3K4me1  10  1.76004574 0.0369204571 0.221522742
## H3K4me3    H3K4me3   6 -0.01026780 0.5159069895 1.000000000
```

Or a visualization of the result can be be outputted by:

```r
VSEplot(bca.vse, las = 2, pch = 20, cex = 1, cex.main = 0.6, padj = 0.05, main = "BCa AVS in MCF7 genomi
```

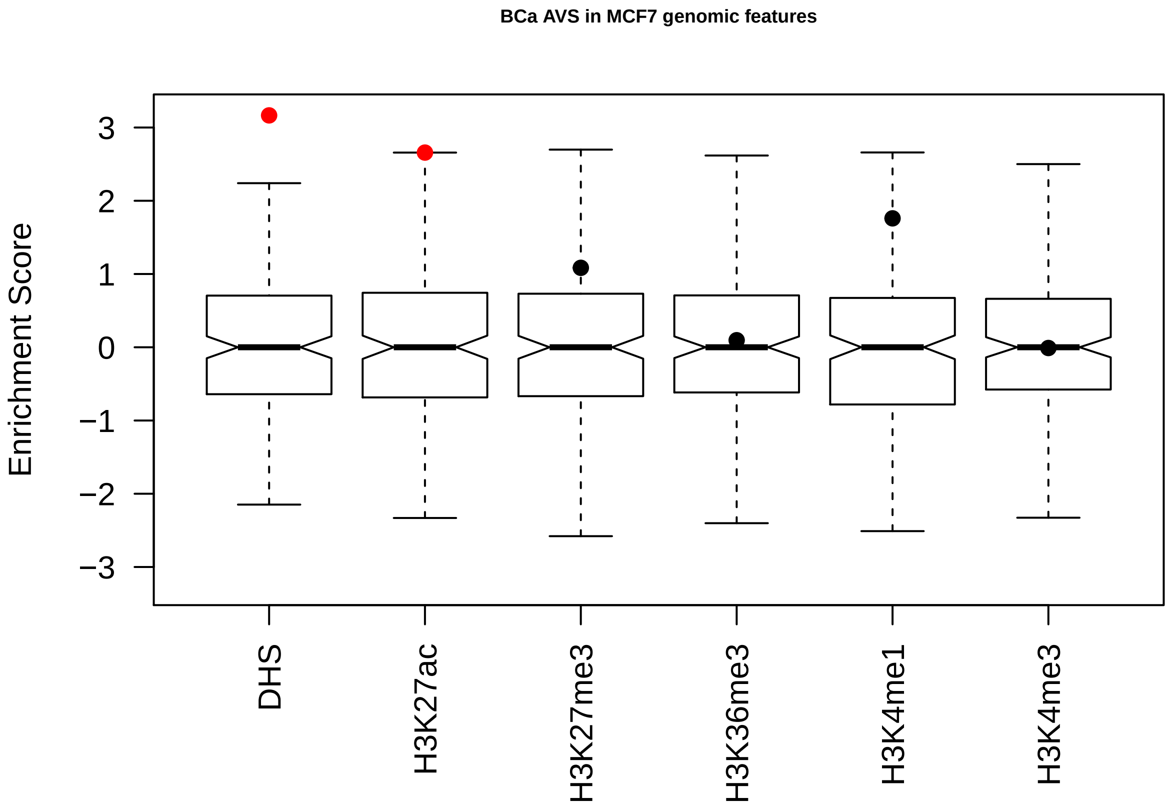

Caution

There are several factors to be considered while using VSE:

1. VSE is sensitive to the number of tagSNPs. From our trial and error tests, too low number of tagSNPs (below 15) provide imprecise result.
2. The quality of ChIP-seq data is very important. We recommend users to confirm the quality of the ChIP-seq data and to only use data that are of good quality to avoid false enrichment. There are tools like ChIPQC or Chillin for quality control of ChIP-seq data.
3. Make sure that you use r2 cutoff of 0.8 when calculating the SNPs in LD.

4. Make sure that the input files are from the same genomic build (e.g., both SNPs and genomic features are in Hg19).
5. Null size is a critical factor for reproducible results. Higher the null size better the normalcy of the distribution. 500 is recommended though it was longer time to compute. It is wiser to compute large MRVS once and save.

Pre-computed MRVSs

Lung cancer AVS: `http://www.hansenhelab.org/VSE/LCa-AVS.RData` Lung cancer MRVSs: `http://www.hansenhelab.org/`[illegible] Colorectal cancer AVS: `http://www.hansenhelab.org/VSE/CCa-AVS.RData` Colorectal cancer MRVSs: `http://www.hansenhelab.org/VSE/CCa-MRVS-200.RData` Prostate cancer AVS: `http://www.hansenhelab.org/VSE/PCa-A`[illegible] Prostate cancer MRVSs: `http://www.hansenhelab.org/VSE/PCa-MRVS-200.RData`

To download and load:

```
download.file(url, destfile=file, method = "curl")
load(file)
```
